# The strain-generated electrical potential in cartilaginous tissues: a role for piezoelectricity

**DOI:** 10.1007/s12551-021-00779-9

**Published:** 2021-02-19

**Authors:** Philip Poillot, Christine L. Le Maitre, Jacques M. Huyghe

**Affiliations:** 1 Bernal Institute, University of Limerick, Limerick, Ireland; 2 Biomolecular Sciences Research Centre, Sheffield Hallam University, Sheffield, UK; 3 Department of Mechanical Engineering, Eindhoven University of Technology, Eindhoven, The Netherlands

**Keywords:** Intervertebral disc, Cartilage, Electrical potential, Streaming potential, Piezoelectricity, Mechanotransduction

## Abstract

The strain-generated potential (SGP) is a well-established mechanism in cartilaginous tissues whereby mechanical forces generate electrical potentials. In articular cartilage (AC) and the intervertebral disc (IVD), studies on the SGP have focused on fluid- and ionic-driven effects, namely Donnan, diffusion and streaming potentials. However, recent evidence has indicated a direct coupling between strain and electrical potential. Piezoelectricity is one such mechanism whereby deformation of most biological structures, like collagen, can directly generate an electrical potential. In this review, the SGP in AC and the IVD will be revisited in light of piezoelectricity and mechanotransduction. While the evidence base for physiologically significant piezoelectric responses in tissue is lacking, difficulties in quantifying the physiological response and imperfect measurement techniques may have underestimated the property. Hindering our understanding of the SGP further, numerical models to-date have negated ferroelectric effects in the SGP and have utilised classic Donnan theory that, as evidence argues, may be oversimplified. Moreover, changes in the SGP with degeneration due to an altered extracellular matrix (ECM) indicate that the significance of ionic-driven mechanisms may diminish relative to the piezoelectric response. The SGP, and these mechanisms behind it, are finally discussed in relation to the cell response.

## Introduction

Cartilaginous tissues, such as articular cartilage (AC) and the intervertebral disc (IVD), are known to remodel in response to a variety of stresses (Grodzinsky et al. 2000; Fearing et al. 2018). Cells embedded in the extracellular matrix (ECM) sense such forces and respond through a milieu of signalling pathways to produce anabolic or catabolic effects. This response is highly dependent on the type, magnitude and frequency of the applied force, as well as the matrix which the cells are embedded in (Buschmann et al. 1995; Neidlinger-Wilke et al. 2006; Korecki et al. 2009; Zhang et al. 2011). Altered biomechanics, leading to a catabolic cell response, can disrupt this important homeostatic control mechanism.

The IVD is highly susceptible to degeneration, a condition which is strongly implicated in low back pain (Luoma et al. 2000, 2016). The primary function of the IVD is to transmit spinal loads while providing for flexibility. As such, the mechanobiology of the IVD have been identified as key processes in maintaining tissue or inducing degeneration (Vergroesen et al. 2015), while the mechanotransduction pathways involved remain poorly understood (Fearing et al. 2018; Molladavoodi et al. 2020). Similarly, biomechanical factors play a critical role in the maintenance or degeneration of AC, while the precise relationship between altered biomechanics and inflammation is not well known (Guilak 2012; Martínez-Moreno et al. 2019).

Many mechanotransduction pathways in AC and the IVD have been elucidated. Most known mechanosensors in both tissues are thought to be signalled by deformations of the local extracellular matrix (ECM) or pericellular matrix (PCM) (Zhao et al. 2020). These lead to a cascade of downstream intracellular signalling to alter transcription in the nucleus, modify gene expression and synthesise protein effectors to repair or degrade the ECM. PIEZO channels (Lee et al. 2014), TRPV channels (O’Conor et al. 2014) and integrins (Le Maitre et al. 2009) are some of the most well-studied mechanosensors in cartilaginous tissue that can be signalled by compression, tensile stretch, hydrostatic pressure, shear stress or other such mechanical cues. The mechanotransduction pathways involved in IVD cells and chondrocytes have been reviewed in-depth elsewhere and thus is beyond the scope of this review (Sanchez-Adams et al. 2014; Fearing et al. 2018).

A well-established mechanism in these tissues in response to loading, distinct from mechanical cues, is the strain-generated potential (SGP). This electrophysiological mechanism was first observed in bone, whereby the mechanical strain of bone generated differential electrical potentials (Friedenberg and Brighton 1966). The SGPs were first attributed to piezoelectricity, whereby noncentrosymmetric molecules generate a charge upon deformation, though streaming potentials, a fluid- and ion-driven electrical mechanism, drew greater interest in subsequent years. SGPs were later observed across several types of cartilage (Bassett and Pawluk 1972), widening the field of stress-induced remodelling to soft tissues. The observation that chondrocytes can respond to loading through voltage-gated ion channels (Tanaka et al. 2005; Mouw et al. 2007; Srinivasan et al. 2015) has further highlighted the role of the SGP in AC and IVD mechanotransduction. The highly hydrated nature of these tissues has made Donnan, diffusion and streaming potentials the dominant topic of study in the mechanisms behind the SGP.

Despite a greater focus on ionic-driven SGPs, piezoelectricity continues to be studied at all hierarchal levels of biological structures, from macro-scale lung tissue (Jiang et al. 2017) to micro-scale amino acids (Guerin et al. 2018). The demonstration of piezoelectric effects has become so widespread that it can be assumed that most biological structures have inherent piezoelectric properties (Guerin et al. 2019). However, the physiological relevance of these effects remains in doubt, largely due to the magnitude of observed piezoelectric responses in comparison to the full electrical potential generated in a loaded tissue.

This review will focus on the SGP in cartilaginous tissue (specifically AC and IVD) and the mechanisms behind it: namely Donnan, diffusion, streaming potentials and piezoelectricity. Both experimental and numerical investigations are discussed in light of the relevant contribution of these mechanisms to the SGP. While many studies have argued that streaming and diffusion potential dominate, an argument is presented herein for the physiological relevance of piezoelectricity in generating and modifying the SGP.

## Ionic-driven mechanisms

### Donnan potentials

**Fig. 1:**
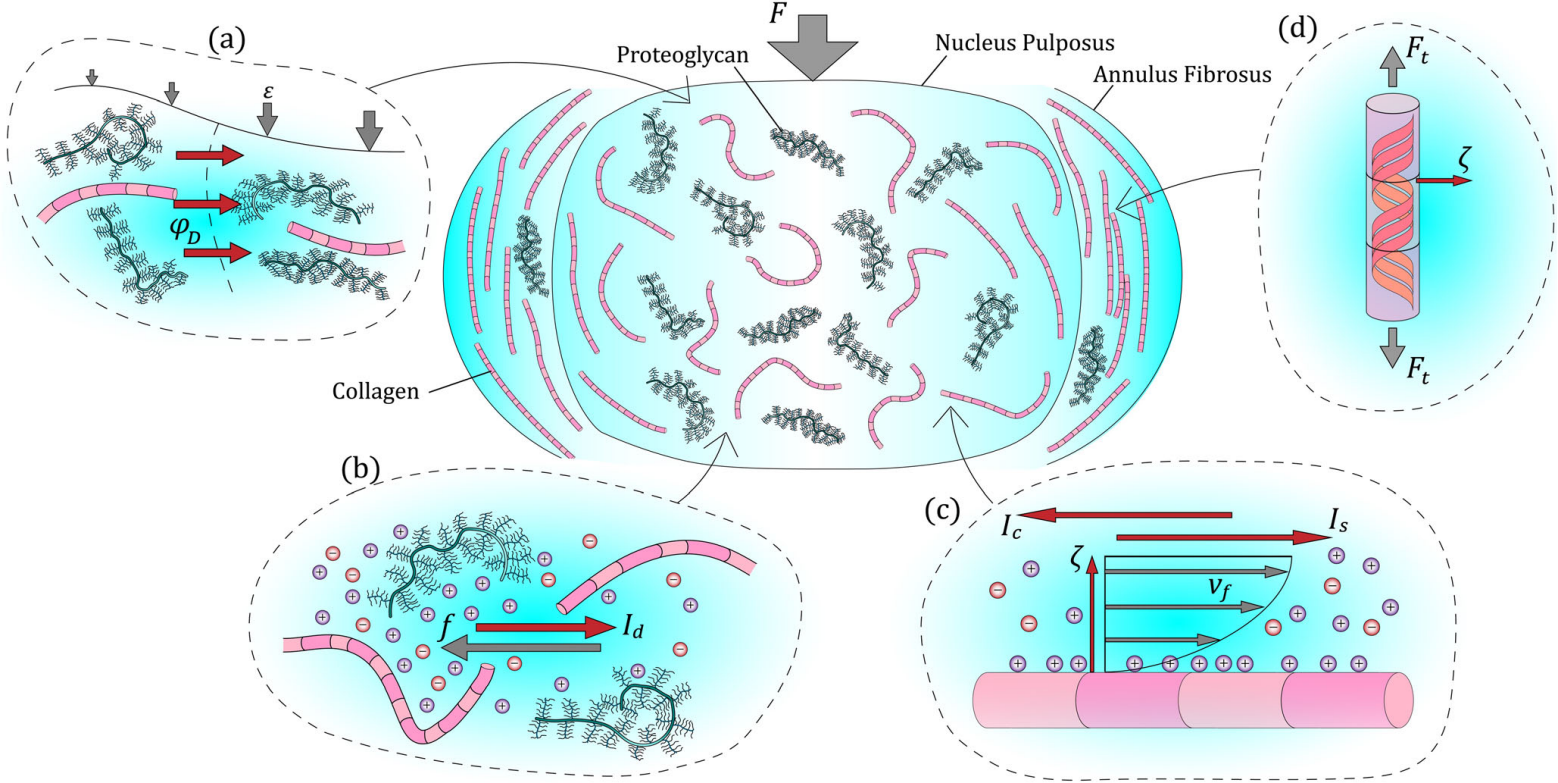
The IVD under a compressive load (*F*), highlighting the major ECM macromolecules of collagen fibres and proteoglycans, with detail views on the different mechanisms of the SGP. **a** Donnan potential. Non-uniform strain (*ε*) of the matrix yields a higher concentration of FCD on the right-hand side. This generates a Donnan potential (*φ*_*D*_) from areas of less-concentrated FCD to areas of higher-concentrated FCD, separated here by the dashed line. **b** Diffusion potential. Loading generates net fluid flow (*f*) that pulls the distributed charged ions (denoted by “+” and “−” symbols) with it. This generates a diffusion current (*I*_*d*_) in the opposite direction to fluid flow by disrupting the balance of positive and negative ions in the ECM. **c** Streaming potential. The double-layer of charged ions by each macromolecule is disrupted by the convection of ions. As shown by the fluid velocity profile (*v*_*f*_), the outer charged layer is disrupted, generating a streaming current (*I*_*s*_) in the direction of fluid flow and an opposing conduction current (*I*_*c*_). The differential disruption of the double-layer generates a zeta potential (*ζ*) acting from the molecule surface to the fluid bulk. **d** Piezoelectricity. The noncentrosymmetric structure of collagen, owing to its non-uniform distribution of charged groups along the triple helix, generates dipole moments when subject to strain (as shown by tensile forces (*F*_*t*_) here in the AF). This generates a net zeta potential (*ζ*) acting out from the surface

Cartilaginous tissues must follow the law of electroneutrality; that is, the tissue must carry a zero net charge. ECM macromolecules in the IVD and AC, however, possess negatively charged surfaces which accumulatively are known as the fixed-charge density (FCD). Proteoglycans are the primary component in cartilaginous tissue that contribute to the FCD. Glycosaminoglycan (GAG) chains, covalently bonded to the proteoglycan monomer, contain negatively charged sulphate and carboxyl groups, creating a net negative surface charge on the GAGs (Frank and Grodzinsky 1987). The FCD attracts positively charged counter-ions, such as calcium and sodium, which become bound within the ECM to preserve electroneutrality. The resulting imbalance of mobile ions between the hydrated matrix and the surrounding solution generates a swelling pressure, or Donnan osmotic pressure, that is balanced with the constraining forces in the collagen network and external loads (Maroudas 1968; Mow and Guo 2002). The associated difference in electrical potential between matrix and surrounding fluid is the Donnan potential, which varies with local FCD. A Donnan potential can also be created within the tissue by a difference in FCD between two local points (Fig. 1a), whereby water and ions may flow between points but the FCD cannot (Huyghe and Bovendeerd 2004).

### Diffusion potentials

Diffusion potentials are also dependent on the FCD. The mobile ions bound within tissue matrix have a tendency to diffuse to areas of lower concentration, but the if the FCD is nonuniform, such as in cartilage (Maroudas 1968) and across the IVD (Urban and Maroudas 1979), Donnan potential gradients will create areas of ion concentration gradients even at equilibrium. A diffusion potential is generated when displacement of the local FCD and ion concentration occurs, driving ionic diffusion. This diffusion potential acts in the direction opposite to diffusion (Fig. 1b) (Lai et al. 2000).

The IVD is subjected to a diurnal loading pattern throughout the day, whereby approximately 25% of tissue fluid is exuded by the pressure of external forces during daytime activity and subsequently imbibed during rest at night (Sivan et al. 2006). This exudation of water increases FCD concentrations and ECM osmolarity during the day. Both Donnan and diffusion potentials are highly dependent on FCD and water content and are therefore susceptible to significant changes with this diurnal cycle of loading/unloading. AC similarly undergoes a diurnal cycle of fluid exudation/recovery of water content, where diurnal strains can be expected to range between 1 and 6%, dependent on AC location (Coleman et al. 2013).

### Streaming potentials

Streaming potential is an electrokinetic phenomenon observed in connective tissue, caused by convective movement of ions bound by the FCD. The inverse of this, electro-osmosis, generates fluid flow by application of an electric field. Electro-osmosis was first discovered in 1807, as Reuss (1809) observed fluid flow across a porous barrier towards the cathode when subject to an electric field. Streaming potential was later observed by Quincke (1861), by measuring the potential difference at either ends of a tube of flowing water. In relating streaming potential to electro-osmosis, Quincke introduced the concept of the double layer theory. Streaming potentials were first measured in bone by Cerquiglini et al. (1967). These were hypothesized to be part of the stress-induced remodelling process, distinct from piezoelectricity. Measurement of streaming potential in cartilage followed shortly after (Maroudas 1968).

The FCD of connective tissue creates a double layer of electrically bound ions in the interstitium, or fluid-filled spaces in tissue. Adsorbed counter-ions are electrostatically bound to form the inner Stern layer, while a higher concentration of counter-ions than co-ions are weakly held further from the surface to form a second diffuse layer (Eriksson 1974). An electric current, or streaming current, is generated when tissue strain induces convective fluid flow that disrupts the diffuse layer, while the voltage difference is termed the streaming potential (Fig. 1c). With regards to ionic fluid flow through a thin cylindrical channel with charged surfaces, the streaming potential is given by Eq. 1 (Gross and Williams 1982).

1 $$ \varepsilon =\frac{\epsilon \zeta \Delta P}{\sigma \eta} $$

Where *ε* is the electrical potential, *ϵ* is the dielectric constant of the solution, Δ*P* is the pressure difference through the channel, *σ* is the conductivity of the solution and *η* is the solution viscosity. The zeta potential, designated by *ζ*, refers to the electrical potential created between the surface and fluid bulk.

## The strain-generated potential

When cartilaginous tissues such as the IVD are subject to a load (Fig. 1), the SGP is generated through Donnan, diffusion and streaming potentials, as well as through piezoelectricity as discussed in this review. Under a load, interstitial fluid is forced through pores and disrupts the balance of counter-ions and co-ions throughout the ECM. The initial convective current of fluid disrupts the diffuse double layer by pulling counter-ions away from the surface of molecules. This generates a streaming current in the direction of fluid flow while, through electroneutrality, also creating a conduction current in the opposite direction (Frank and Grodzinsky 1987). The net potential generated by disruption of the double-layer is termed the streaming potential. The applied load generates Donnan potentials acting with streaming potentials, whereby non-uniform deformation of the matrix yields a difference in local FCD (Huyghe et al. 2007). When a streaming current disrupts the local ionic concentration gradients, a diffusion current, and therefore diffusion potential, also acts in opposition to the streaming current (Lai et al. 2000). The net potential created by these mechanisms generates an electrical field and a zeta potential acting out from the surface of the ECM.

## Piezoelectricity

Many tissues have long been known to remodel and heal in response to stress. Wolff’s law accounted for this response in bone, whereby the shape and structure of the tissue adapts to the external mechanical stimulus (Wolff 1986). This relationship has been argued to be electrically driven, largely due to the link between electrical stimulation and fracture healing (Yasuda 1977). These earlier studies postulated that piezoelectricity was the mechanism responsible for these effects, linking stress to remodelling through an electrical response.

Piezoelectricity is a ferroelectric phenomenon, whereby mechanical energy is directly coupled to electrical energy. In structures that lack a centre of symmetry, a mechanical strain can generate an electrical charge by the direct piezoelectric effect (Fig. 1d). Many biological structures are noncentrosymmetric and demonstrate this linear electromechanical coupling, with collagen being the most studied piezoelectric structure among these. Subject to a deformation, polar groups change conformation and reorient in the direction of applied strain, inducing a dipole moment about the axis (Zhou et al. 2016). This perturbation of electroneutrality, occurring at many different sites in the strained molecule, generates a net polarisation at the surface (Stapleton et al. 2017). The inverse piezoelectric effect has also been demonstrated in these materials, whereby an applied electrical field can induce a surface deformation. The electrical displacement, *D*, generated by the direct piezoelectric effect is given by Eq. 2:

2 $$ \left\{D\right\}=\left[d\right]\left\{T\right\}+\left[{\epsilon}^t\right]\left\{E\right\} $$

where [*d*] is the direct piezoelectric effect matrix, *T* is the constant stress field, [*ϵ*^*t*^] is the transpose of the permittivity matrix and *E* is the electric field strength.

Piezoelectricity was first studied in biological tissue in the 1950s (Fukada and Yasuda 1957) and attracted much interest due to the recent discovery, at the time, of the link between stress, electrical stimulation and fracture healing. Many studies followed, attempting to ascertain the relationship between piezoelectricity and bone healing. However, while the theory of piezoelectricity-driven remodelling garnered support in studies of dry bone, several studies disputed this mechanism when bone was studied in a hydrated state (Anderson and Eriksson 1970; Dwyer and Matthews 1970; Johnson et al. 1980). Instead, these studies argued that streaming potentials were the dominant mechanism in mediating the SGP owing to the magnitudes of charge generated by both mechanisms.

While interest in the role of biological piezoelectricity in SGPs diminished until more recent years, the field has intensified on piezoelectric energy harvesting in materials engineering. Inorganic piezoelectric materials, such as zinc oxide, have been widely studied for use as nanosensors (Wang 2004), in-vivo energy harvesters for implant monitoring (Platt et al. 2005) and self-powered nanosystems (Wu et al. 2014). With a view to design drug delivery systems, research has also accelerated on understanding the piezoelectric properties of organic biomolecules (Guerin et al. 2019). However, Ahn and Grodzinsky (2009) proposed a novel mechanism of piezoelectricity in SGPs, in that piezoelectric effects work in conjunction with streaming potentials in bone. This re-ignited interest in understanding biological piezoelectricity, leading to more recent measurements of piezoresponses in tendon (Denning et al. 2012) and intervertebral disc (IVD) (Poillot et al. 2020). Through decades of study, piezoelectricity has been proposed to be an inherent property of most biological structures (Guerin et al. 2019), while the molecule primarily responsible for this effect in bone, and likely connective tissues, is collagen (Halperin et al. 2004). The question remains, however, of the physiological relevance of piezoelectricity in such hydrated tissues when compared with ionic- and fluid-driven phenomena.

## Numerical models

### Triphasic and quadriphasic mixture models

Several different numerical models have been developed to account for ionic-driven SGPs in tissue using continuum mixture theory; that is, every point in the model is occupied simultaneously by each phase of the mixture (solid, fluid, ionic). Mixture theory originates from the work of Truesdell (1957) and Truesdell and Toupin (1960), and attempts to unify all theories involving miscible and immiscible mixtures of solids, fluids and gases. Bowen (1976, 1980, 1982) was the first to demonstrate that classical porous media theories in small deformations (Biot 1941) and large deformation (Biot 1972) can be derived from mixture theory. McCutchen (1959, 1962) describes cartilage as a porous medium, obeying Biot’s theory. Along the same lines, Mow et al. (1980) used mixture theory to model cartilage as a biphasic medium, which treated cartilage as a linear-elastic solid phase of ECM molecules and a fluid phase of viscous interstitial fluid.

This theory was later expanded to a triphasic mixture theory (Lai et al. 1991), whereby the ionic phase was introduced as a second fluid phase to account for the FCD and ionic diffusion. The triphasic theory was the first comprehensive model to account for the ionic phase in soft tissue and was used extensively to model cartilage deformation (Gu et al. 1993, 1997; Mow et al. 1998; Sun et al. 1999). The model was expanded several times, allowing for the modelling of streaming potentials, diffusion potentials and Donnan potentials (Gu et al. 1998).

The triphasic theory was limited, however, in that it neglected electrical fluxes. Huyghe and Janssen (1997) developed a finite deformation quadriphasic theory to overcome these limitations, in which the four phases consist of the solid phase, fluid phase, monovalent cation phase and monovalent anion phase. The quadriphasic theory, like the triphasic theory, has been widely employed to model the behavior of soft porous tissue and gels. These mixture theories utilized the classical Nernst equation (Nernst 1889) to derive the SGP in cartilage from Donnan, diffusion and streaming potentials.

Both the triphasic and quadriphasic theory, however, only account for electrochemical potentials associated with ionic and fluid flow. As evident from those model’s equations, the electrical potential of the tissue or gel depends only on ionic activity and concentration terms, that themselves are modified by local FCD and fluid movement. These models implicitly assume that strain-generated electrical potentials depend only on the movement of ions and fluid in the tissue, neglecting ferroelectric effects such as piezoelectricity.

### Mixed hybrid finite element model

More recently, Yu et al. (2018) developed a mixed hybrid finite element method to model large deformation in hydrogels which employed local mass conservation and calculated fluid flux as an independent variable to better replicate large deformation in porous media. This was modified by Fennell and Huyghe (2020) to reflect experimental data on hydrogel (Roos et al. 2013) and on cartilage (Jin and Grodzinsky 2001) subject to shear. Both Roos et al. (2013) and Jin and Grodzinsky (2001) subjected hydrogel/cartilage to simple shear while altering the bath osmolarity and found that stiffness was dependent on ionic concentration, thereby disobeying classic Donnan theory. The model of Fennell and Huyghe (2020) derived new Donnan equations and found a direct dependence of electrical potential on strain. While previous models used regular Donnan theory to couple Donnan potential to volume change, the experimental evidence and subsequent model shows potential dependence on shape change as well. As shape change occurs immediately upon loading of cartilaginous tissues, this demonstrates a direct, immediate coupling of strain and electrical potential, independent on fluid and ion movement.

The authors of the cartilage experiment (Jin and Grodzinsky 2001), hydrogel experiment (Roos et al. 2013) and numerical model (Fennell and Huyghe 2020) attribute this strain-dependent potential to electrostatic interactions between GAG molecules as they move relative to one another. While such electrostatic interactions are caused by polarization of ions in the double layer relative to the fixed charge, piezoelectric effects may also be the responsible mechanism. In such a scenario of simple shear, piezoelectric molecules could be expected to undergo immediate polarization, independent of fluid flow and volume change. As collagen is primarily a shear-piezoelectric material (Minary-Jolandan and Yu 2009), this response seems plausible in cartilage and the IVD. Furthermore, Fennell and Huyghe (2020) describe the potential difference due to shape change as the order of mV, but only when such shape changes is of the order of 200%. In smaller, physiologically relevant shape change, the potential difference is in the order of nV, which is in the range of piezoresponse measured in tissues and biological molecules.

## Relative contribution to SGPs

### Fluid-driven mechanisms

Many experimental studies have been performed to investigate the SGP in cartilaginous tissues. These have employed confined and unconfined dynamic compression stimuli on AC and IVD tissue samples while measuring the potential difference either within a sample or across a sample (Lee et al. 1981; Frank and Grodzinsky 1987; Garon et al. 2002; Iatridis et al. 2016). Such studies have consistently found an electrical potential of the order of ≈ 1 mV, which has usually been referred to as the “streaming potential” only. In most cases, only numerical models attribute the electrical potential generated in loaded tissue to a combination of streaming, diffusion and Donnan potentials. Indeed, it has been demonstrated that, in soft tissue such as cartilage, the diffusion potential may dominate the streaming potential (Lai et al. 2000).

The GAG-collagen ratio in the healthy NP of the IVD (27:1) is much higher than in the outer AF (1.6:1) or in AC (2:1) (Mwale et al. 2004). As the difference in GAG content between NP and AF is large, the Donnan potential between these two neighboring tissues is likely to be significant. Similarly, as the NP has a high proteoglycan content with low constraining collagen forces, one could speculate that the local FCD could be more easily altered upon loading, leading to a greater Donnan potential in the NP than in the AF or AC. The greater FCD in the NP also indicates a greater streaming and diffusion potentials in the NP than in the AF (Iatridis et al. 2016) and, likely, the AC. Differences in loading, environmental conditions and measurement methods make a precise comparison between studies on the SGP in the IVD and AC impractical.

In degeneration, the magnitude of the SGP is reduced, in correlation with reduced GAG content, in the IVD (Gu et al. 1999; Iatridis et al. 2003) and in AC (Chen et al. 1997; Légaré et al. 2002; Abedian et al. 2013). Concurrently to this loss of GAG and water content in degenerated tissues, more stress upon loading is placed on the solid matrix components (Iatridis et al. 2003). Moreover, the ratio of GAG-collagen in both tissues decreases with degeneration (Mwale et al. 2004). In the NP, aggrecan and collagen II synthesis is diminished while more collagen I is produced. With reduced water content, higher ratios of less-compliant collagen I and a stiffer collagen network owing to increased cross-linking (Duance et al. 1998), loading of the IVD generates higher shear stresses in place of physiological hydrostatic pressure and tension in the NP and AF, respectively (Vergroesen et al. 2015). Similarly in AC, abundant collagen II molecules stiffen in osteoarthritis due to increased cross-linking (Rahmati et al. 2017). As more stress, particularly shear, is born by more collagen molecules, it is likely that the magnitude of piezoelectric responses would be greater, and thus more physiologically relevant to the SGP, in degenerated IVD and AC.

### Piezoelectricity

Despite the evidence for piezoelectricity across most biological structures, no experimental or numerical study has incorporated piezoelectric effects in the electromechanical response of AC or IVD under load. Experimental investigations on piezoelectricity in these hydrated tissues have faced the challenge of isolating piezoelectric effects from fluid-driven electrical effects. The most obvious approach to overcome this is to remove the water content of the tissue being tested, an approach that has traditionally been used to investigate bone piezoelectricity (Fukada and Yasuda 1957; Shamos et al. 1963). While dehydrated bone is not physiological, the much more hydrated nature of cartilaginous tissues makes such an approach even less so in those tissues. Despite this, attempts have been made to isolate and quantify piezoelectric effects in the hydrated IVD (Poillot et al. 2020).

The more recent development of piezoresponse force microscopy (PFM) has made investigations of isolated piezoelectric effects in hydrated tissues more attainable. PFM is a variant of atomic force microscopy, whereby an AC voltage is applied by a conductive probe tip in contact with the sample of interest. The electrical field generated results in surface deformation, via the inverse piezoelectric effect, which is measured by deflection of the cantilevered probe. This approach has been used to study piezoelectricity across wide length scales, including bone (Halperin et al. 2004), tendon (Denning et al. 2012), IVD (Poillot et al. 2020), collagen (Minary-Jolandan and Yu 2009), elastin (Liu et al. 2014) and amino acids (Guerin et al. 2018). The magnitude of isolated piezoresponse in soft tissues, however, is of a much lower magnitude than the net potential, of ≈ 1 pC/N, or 1 nV in the loaded IVD.

There are two hypotheses as to how the relatively small recordings of piezoelectricity in tissues may be physiologically relevant. The first is proposed by Minary-Jolandan and Yu (2009), who demonstrate that collagen is primarily a shear-piezoelectric structure, while longitudinal piezoelectricity, the response usually measured by PFM and dehydrated macro-scale measurements, is almost negligible. They calculate that, subject to physiological loading, type I collagen fibrils could generate local shear piezoelectric charges of the order of mV. In differentiating isolated amino-acids, Guerin et al. (2018) confirmed unusually high shear-piezoelectric responses of glycine. The second hypothesis is proposed by Ahn and Grodzinsky (2009), who argue that piezoelectric effects work in conjunction with streaming potentials by altering the zeta potential acting out from the collagen surface and modifying the streaming current of ions. Both of these mechanisms may act in tandem, whereby a high shear-piezoelectric response modifies the zeta potential and thus the streaming potential through the tissue.

## Cell response

As mentioned earlier, resident cells sense and respond to loading through many different mechanotransduction pathways. The cell response to the SGP, distinct from response to the mechanical load itself, is of particular interest here. Voltage-gated ion channels (VGICs) are one such mechanosensing pathway that are implicated in this. Chondrocytes in large mammals have a resting membrane potential, of about – 10 mV (Lewis et al. 2011), that allow for a cell response to electrical potential changes. VGICs channels, such as voltage-gated calcium or potassium channels, have been widely studied in this role, as they can mediate a rapid influx of that specific ion upon cell depolarisation to launch the cell response (Matta et al. 2015). These VGICs have been shown to partly mediate a variety of mechanoresponses in chondrocytes, such as protein expression in response to cyclic tensile strain (Tanaka et al. 2005), protein and GAG synthesis in response to static/dynamic compression (Mouw et al. 2007) and aggrecan synthesis in response to shear strain (Srinivasan et al. 2015). Such differential responses to a variety of stresses are likely due to the generation of different SGPs. No such investigations have been performed on VGICs, or other electrically-driven pathways, in IVD cells.

In osteoarthritis, chondrocytes respond differently to loading with different mechanotransduction pathways involved (Millward-Sadler and Salter 2004; Lohberger et al. 2019). IVD cells similarly respond differently in degeneration, and through different pathways (Le Maitre et al. 2009; Gilbert et al. 2013). In relation to the SGP, VGIC mechanotransduction is altered in osteoarthritic AC (Srinivasan et al. 2015), as is the basic chondrocyte electrophysiological response (Millward-Sadler et al. 2000). This altered mechanotransduction is thought to be driven by a change to the cell phenotype as well as changes in the ECM. Indeed, as was discussed earlier, degenerated IVD and AC have been shown to generate an altered SGP under loading. As the mechanotransduction pathway between the SGP and healthy/degenerate cells is so complex, much more work needs to be done to elucidate the relationship between the SGP, the composition of the ECM and the cell response.

## Conclusion

Difficulties in quantifying the piezoelectric response of cartilage and the IVD has hampered the evidence for its physiological relevance. The reported small values of piezoelectricity may be an underestimation at the macro-scale, as higher shear-piezoelectric responses may be more relevant in modifying other mechanisms in the SGP, particularly the streaming potential. This may be particularly true for degenerated tissues, where a reduced water content, stiffer matrix and altered biomechanics all support a greater role for piezoelectricity. The relevance of the SGP to mechanotransduction is not as clear; a definitive link has been demonstrated but the precise relationship between the magnitude of Donnan, diffusion or streaming potential and the cell response, likely through VGICs, is yet to be elucidated. Further, the relevance of piezoelectricity to the cell response has only been inferred. Numerical models, that have so far neglected ferroelectric effects, could be of great use in this regard, particularly as new evidence supports a direct link between strain and the SGP. Only further investigations, particularly in isolated biological components and comprehensive numerical models, may elucidate the true physiological significance of piezoelectricity.

## Data Availability

Not applicable.

## References

[CR1] Abedian R, Willbold E, Becher C, Hurschler C (2013). In vitro electro-mechanical characterization of human knee articular cartilage of different degeneration levels: a comparison with ICRS and Mankin scores. J Biomech.

[CR2] Ahn AC, Grodzinsky AJ (2009). Relevance of collagen piezoelectricity to “ Wolff ’ s Law ”: a critical review. Med Eng Phys.

[CR3] Anderson JC, Eriksson C (1970). Piezoelectric Properties of Dry and Wet Bone. Nature.

[CR4] Bassett CAL, Pawluk RJ (1972). Electrical behavior of cartilage during loading. Science.

[CR5] Biot MA (1972). Theory of Finite Deformations of Porous Solids. Indiana Univ Mathematics J.

[CR6] Biot MA (1941). General theory of three-dimensional consolidation. J Appl Phys.

[CR7] Bowen RM, Eringen AC (1976). Theory of Mixtures. Continuum Physics.

[CR8] Bowen RM (1980). Incompressible porous media models by use of the theory of mixtures. Int J Eng Sci.

[CR9] Bowen RM (1982). Porous media model formulations by the theory of mixtures. NATO ASI Ser E Appl Sci.

[CR10] Buschmann MD, Gluzband YA, Grodzinsky AJ, Hunziker EB (1995). Mechanical compression modulates matrix biosynthesis in chondrocyte/agarose culture. J Cell Sci.

[CR11] Cerquiglini S, Cignitti M, Marchetti M, Salleo A (1967). On the origin of electrical effects produced by stress in the hard tissues of living organisms. Life Sci.

[CR12] Chen AC, Nguyen TT, Sah RL (1997). Streaming potentials during the confined compression creep test of normal and proteoglycan-depleted cartilage. Ann Biomed Eng.

[CR13] Coleman JL, Widmyer MR, Leddy HA (2013). Diurnal variations in articular cartilage thickness and strain in the human knee. J Biomech.

[CR14] Denning D, Abu-Rub MT, Zeugolis DI (2012). Electromechanical properties of dried tendon and isoelectrically focused collagen hydrogels. Acta Biomater.

[CR15] Duance VC, Crean JKG, Sims TJ (1998). Changes in collagen cross-linking in degenerative disc disease and scoliosis. Spine.

[CR16] Dwyer NSJP, Matthews B (1970). The electrical response to stress in dried, recently excised, and living bone. Injury.

[CR17] Eriksson C (1974). Streaming potentials and other water-dependent effects in mineralized tissues. Ann N Y Acad Sci.

[CR18] Fearing BV, Hernandez PA, Setton LA, Chahine NO (2018) Mechanotransduction and cell biomechanics of the intervertebral disc. JOR Spine. 10.1002/jsp2.1026 10.1002/jsp2.1026 PMC6296470 30569032

[CR19] Fennell E, Huyghe JM (2020) A three-dimensional mechanoelectrochemical material model of mechanosensing hydrogels. Mater Des. 10.1016/j.matdes.2020.109340

[CR20] Frank EH, Grodzinsky AJ (1987) Cartilage electromechanics-I. Electrokinetic transduction and the effects of electrolyte pH and ionic strength. J Biomech 20. 10.1016/0021-9290(87)90282-X 10.1016/0021-9290(87)90282-x 3611137

[CR21] Friedenberg ZB, Brighton C (1966). Biolectric potentials in bone. J Bone Joint Surg.

[CR22] Fukada E, Yasuda I (1957). On the piezoelectric effect of bone. J Phys Soc Jpn.

[CR23] Garon M, Légaré A, Guardo R (2002). Streaming potentials maps are spatially resolved indicators of amplitude, frequency and ionic strength dependant responses of articular cartilage to load. J Biomech.

[CR24] Gilbert HTJ, Nagra NS, Freemont AJ et al (2013) Integrin—dependent mechanotransduction in mechanically stimulated human annulus fibrosus cells: evidence for an alternative mechanotransduction pathway operating with degeneration. PLoS ONE 8. 10.1371/journal.pone.0072994 10.1371/journal.pone.0072994 PMC3764176 24039840

[CR25] Grodzinsky AJ, Levenston ME, Jin M, Frank EH (2000). Cartilage tissue remodeling in response to mechanical forces. Annu Rev Biomed Eng.

[CR26] Gross D, Williams WS (1982). Streaming potential and the electromechanical response of physiologically-moist bone. J Biomech.

[CR27] Gu WY, Lai WM, Mow VC (1997). A triphasic analysis of negative osmotic flows through charged hydrated soft tissues. J Biomech.

[CR28] Gu WY, Lai WM, Mow VC (1993). Transport of fluid and ions through a porous-permeable charged-hydrated tissue, and streaming potential data on normal bovine articular cartilage. J Biomech.

[CR29] Gu WY, Lai WM, Mow VC (1998). A mixture theory for charged-hydrated soft tissues containing ivjulti-electrolytes: Passive transport and swelling behaviors. J Biomech Eng.

[CR30] Gu WY, Mao XG, Rawlins BA (1999). Streaming potential of human lumbar anulus fibrosus is anisotropic and affected by disc degeneration. J Biomech.

[CR31] Guerin S, Stapleton A, Chovan D (2018). Control of piezoelectricity in amino acids by supramolecular packing. Nat Mater.

[CR32] Guerin S, Tofail SAM, Thompson D (2019). Organic piezoelectric materials: milestones and potential. NPG Asia Mater.

[CR33] Guilak F (2012). Biomechanical factors in osteoarthritis. Best Pract Res Clin Rheumatol.

[CR34] Halperin C, Mutchnik S, Agronin A (2004). Piezoelectric effect in human bones studied in nanometer scale. Nano Lett.

[CR35] Huyghe JM, Bovendeerd PHM, Loret B, Huyghe JM (2004). Chemo-mechanical couplings in porous media geomechanics and biomechanics. Chemo-Mechanical Couplings in Porous Media Geomechanics and Biomechanics.

[CR36] Huyghe JM, Janssen JD (1997). Quadriphasic mechanics of swelling incompressible porous media. Int J Eng Sci.

[CR37] Huyghe JM, Molenaar MM, Baajens FPT (2007). Poromechanics of compressible charged porous media using the theory of mixtures. J Biomech Eng.

[CR38] Iatridis JC, Laible JP, Krag MH (2003) Influence of fixed charge density magnitude and distribution on the intervertebral disc: applications of a poroelastic and chemical electric (PEACE) model. J Biomech Eng 125. 10.1115/1.1533804 10.1115/1.1537190 12661193

[CR39] Iatridis JC, Stokes IAF, Gardner-morse MG, Laible JP (2016) Potentials of human intervertebral disk motion segments under dynamic axial. 131:11–13. 10.1115/1.3005164 10.1115/1.3005164 PMC2757255 19154065

[CR40] Jiang P, Yan F, Esfahani EN, Xie S et al (2017) Electromechanical coupling of murine lung tissues probed by Piezoresponse Force Microscopy. ACS Biomater Sci Eng. 10.1021/acsbiomaterials.7b00107 10.1021/acsbiomaterials.7b00107 33429664

[CR41] Jin M, Grodzinsky AJ (2001). Effect of Electrostatic Interactions between glycosaminoglycans on the shear stiffness of cartilage : a molecular model and experiments. Macromolecules.

[CR42] Johnson MW, Chakkalakal DA, Harper RA, Katz JL (1980). Comparison of the electromechanical effects in wet and dry bone. J Biomech.

[CR43] Korecki CL, Kuo CK, Tuan RS, Iatridis JC (2009). Intervertebral disc cell response to dynamic compression is age and frequency dependent. J Orthop Res.

[CR44] Lai WM, Hou JS, Mow VC (1991). A triphasic theory for the swelling and deformation behaviors of articular cartilage. J Biomech Eng.

[CR45] Lai WM, Mow VC, Sun DD, Ateshian GA (2000). On the electric potentials inside a charged soft hydrated biological tissue: streaming potential versus diffusion potential. J Biomech Eng.

[CR46] Le Maitre CL, Frain J, Millward-Sadler J (2009). Altered integrin mechanotransduction in human nucleus pulposus cells derived from degenerated discs. Arthritis Rheum.

[CR47] Lee RC, Frank EH, Grodzinsky AJ, Roylance DK (1981). Oscillatory compressional behavior of articular cartilage and its associated electromechanical properties. J Biomech Eng.

[CR48] Lee W, Leddy HA, Chen Y (2014). Synergy between Piezo1 and Piezo2 channels confers high-strain mechanosensitivity to articular cartilage. Proc Natl Acad Sci U S A.

[CR49] Légaré A, Garon M, Guardo R (2002). Detection and analysis of cartilage degeneration by spatially resolved streaming potentials. J Orthop Res.

[CR50] Lewis R, Asplin KE, Bruce G (2011). The role of the membrane potential in chondrocyte volume regulation. J Cell Physiol.

[CR51] Liu Y, Cai HL, Zelisko M et al (2014) Ferroelectric switching of elastin. Proc Natl Acad Sci U S A 111. 10.1073/pnas.1402909111 10.1073/pnas.1402909111 PMC4103339 24958890

[CR52] Lohberger B, Kaltenegger H, Weigl L (2019). Mechanical exposure and diacerein treatment modulates integrin-FAK-MAPKs mechanotransduction in human osteoarthritis chondrocytes. Cell Signal.

[CR53] Luoma K, Riihimäki H, Luukkonen R (2000). Low back pain in relation to lumbar disc degeneration. Spine.

[CR54] Luoma K, Vehmas T, Kerttula L (2016). Chronic low back pain in relation to Modic changes, bony endplate lesions, and disc degeneration in a prospective MRI study. Eur Spine J.

[CR55] Maroudas A (1968). Physicochemical Properties of Cartilage in the light of ion exchange theory. Biophys J.

[CR56] Martínez-Moreno D, Jiménez G, Gálvez-Martín P (2019). Cartilage biomechanics: a key factor for osteoarthritis regenerative medicine. Biochim Biophys Acta Mol basis Dis.

[CR57] Matta C, Zákány R, Mobasheri A (2015) Voltage-dependent calcium channels in chondrocytes: roles in health and disease. Curr Rheumatol Rep 17. 10.1007/s11926-015-0521-4 10.1007/s11926-015-0521-4 25980668

[CR58] McCutchen CW (1962). The frictional properties of animal joints. Wear.

[CR59] McCutchen CW (1959). Mechanism of animal joints: sponge-hydrostatic and weeping bearings. Nature.

[CR60] Millward-Sadler SJ, Salter DM (2004). Integrin-dependent signal cascades in chondrocyte mechanotransduction. Ann Biomed Eng.

[CR61] Millward-Sadler SJ, Wright MO, Lee HS (2000). Altered electrophysiological responses to mechanical stimulation and abnormal signalling through α5β1 integrin in chondrocytes from osteoarthritic cartilage. Osteoarthr Cartil.

[CR62] Minary-Jolandan M, Yu MF (2009) Nanoscale characterization of isolated individual type i collagen fibrils: Polarization and piezoelectricity. Nanotechnology 20. 10.1088/0957-4484/20/8/085706 10.1088/0957-4484/20/8/085706 19417467

[CR63] Molladavoodi S, McMorran J, Gregory D (2020). Mechanobiology of annulus fibrosus and nucleus pulposus cells in intervertebral discs. Cell Tissue Res.

[CR64] Mouw JK, Imler SM, Levenston ME (2007). Ion-channel regulation of chondrocyte matrix synthesis in 3D culture under static and dynamic compression. Biomech Model Mechanobiol.

[CR65] Mow VC, Ateshian GA, Lai WM, Gu WY (1998) Effects of fixed charges on the stress- relaxation behavior of hydrated soft tissues in a confined compression problem. Int J Solids Struct 35:4945–4962. 10.1016/S0020-7683(98)00103-6

[CR66] Mow VC, Guo XE (2002). Mechano-electrochemical properties of articular cartilage: their inhomogeneities and anisotropies. Annu Rev Biomed Eng.

[CR67] Mow VC, Kuei SC, Lai WM, Armstrong CG (1980). Biphasic creep and stress relaxation of articular cartilage in compression: Theory and experiments. J Biomech Eng.

[CR68] Mwale F, Roughley P, Antoniou J (2004). Distinction between the extracellular matrix of the nucleus pulposus and hyaline cartilage: A requisite for tissue engineering of intervertebral disc. Eur Cells Mater.

[CR69] Neidlinger-Wilke C, Würtz K, Urban JPG (2006). Regulation of gene expression in intervertebral disc cells by low and high hydrostatic pressure. Eur Spine J.

[CR70] Nernst W (1889). Die elektromotorische Wirksamkeit der Jonen. Z Phys Chem.

[CR71] O’Conor CJ, Leddy HA, Benefield HC (2014). TRPV4-mediated mechanotransduction regulates the metabolic response of chondrocytes to dynamic loading. Proc Natl Acad Sci U S A.

[CR72] Platt SR, Farritor S, Garvin K, Haider H (2005). The use of piezoelectric ceramics for electric power generation within orthopedic implants. IEEE/ASME Trans Mechatr.

[CR73] Poillot P, O’Donnell J, O’Connor DT (2020). Piezoelectricity in the Intervertebral disc. J Biomech.

[CR74] Quincke G (1861). Ueber die Fortführung materieller Theilchen durch strömende Elektricität. Ann Phys.

[CR75] Rahmati M, Nalesso G, Mobasheri A, Mozafari M (2017). Aging and osteoarthritis: central role of the extracellular matrix. Ageing Res Rev.

[CR76] Reuss FF (1809). Notice sur un nouvel effet de l’électricité galvanique. Mem Soc Imper Nat Moscou.

[CR77] Roos RW, Petterson R, Huyghe JM (2013) Confined compression and torsion experiments on a pHEMA gel in various bath concentrations:617–626. 10.1007/s10237-012-0429-0 10.1007/s10237-012-0429-0 22926832

[CR78] Sanchez-Adams J, Leddy HA, McNulty AL (2014). The mechanobiology of articular cartilage: bearing the burden of osteoarthritis. Curr Rheumatol Rep.

[CR79] Shamos MH, Lavine LS, Shamos MI (1963). Piezoelectric effect in bone. Nature.

[CR80] Sivan S, Neidlinger-Wilke C, Würtz K (2006). Diurnal fluid expression and activity of intervertebral disc cells. Biorheology.

[CR81] Srinivasan PP, Parajuli A, Price C (2015). Inhibition of T-type voltage sensitive calcium channel reduces load-induced OA in mice and suppresses the catabolic effect of bone mechanical stress on chondrocytes. PLoS ONE.

[CR82] Stapleton A, Noor MR, Sweeney J et al (2017) The direct piezoelectric effect in the globular protein lysozyme. Appl Phys Lett 111. 10.1063/1.4997446

[CR83] Sun DN, Gu WY, Guo XE (1999). A mixed finite element formulation of triphasic mechano-electrochemical theory for charged, hydrated biological soft tissues. Int J Numer Methods Eng.

[CR84] Tanaka N, Ohno S, Honda K (2005). Cyclic mechanical strain regulates the pthrp expression\rin cultured chondrocytes via activation of the ca2+ channel. J Dent Res.

[CR85] Truesdell CA (1957) Sulle basi della termomeccania. Rand Lincei 22:1158–1166

[CR86] Truesdell C, Toupin R (1960) The Classical Field Theories:226–858. 10.1007/978-3-642-45943-6_2

[CR87] Urban JPG, Maroudas A (1979). The measurement of fixed charged density in the intervertebral disc. BBA Gen Sub.

[CR88] Vergroesen PPA, Kingma I, Emanuel KS (2015). Mechanics and biology in intervertebral disc degeneration: A vicious circle. Osteoarthr Cartil.

[CR89] Wang ZL (2004). Zinc oxide nanostructures: Growth, properties and applications. J Phys Condens Matter.

[CR90] Wolff J (1986). The Law of Bone Remodelling.

[CR91] Wu W, Wang L, Li Y (2014). Piezoelectricity of single-atomic-layer MoS2 for energy conversion and piezotronics. Nature.

[CR92] Yasuda I (1977). The classic: fundamental aspects of fracture treatment by Iwao Yasuda, reprinted from J. Kyoto Med. Soc., 4:395-406, 1953. Clin Orthop Relat Res.

[CR93] Yu C, Malakpoor K, Huyghe JM (2018). A three-dimensional transient mixed hybrid finite element model for superabsorbent polymers with strain dependent permeability. Soft Matter.

[CR94] Zhang YH, Zhao CQ, Jiang LS, Dai LY (2011). Substrate stiffness regulates apoptosis and the mRNA expression of extracellular matrix regulatory genes in the rat annular cells. Matrix Biol.

[CR95] Zhao Z, Li Y, Wang M (2020). Mechanotransduction pathways in the regulation of cartilage chondrocyte homoeostasis. J Cell Mol Med.

[CR96] Zhou Z, Qian D, Minary-Jolandan M (2016). Molecular mechanism of polarization and piezoelectric effect in super-twisted collagen. ACS Biomater Sci Eng.

